# Day -1 CD34+ Cells and Platelet Count Predict the Number of Apheresis in Poor-Mobilizer Patients Rescued by Plerixafor

**DOI:** 10.3390/jcm12020618

**Published:** 2023-01-12

**Authors:** Caterina Giovanna Valentini, Claudio Pellegrino, Rossana Putzulu, Matteo Bonanni, Giuseppina Massini, Nicoletta Orlando, Franca Forni, Maria Bianchi, Nicola Piccirillo, Luciana Teofili

**Affiliations:** 1 Dipartimento di Diagnostica per Immagini, Radioterapia Oncologica ed Ematologia, Fondazione Policlinico Universitario “A. Gemelli” IRCCS, I-00168 Rome, Italy; 2 Sezione di Ematologia, Dipartimento di Scienze Radiologiche ed Ematologiche, Università Cattolica del Sacro Cuore, I-00168 Rome, Italy

**Keywords:** plerixafor, autologous hematopoietic stem cell transplantation, CD34+ cells, poor mobilization, predictive factors

## Abstract

Plerixafor is widely used as up-front treatment with G-CSF to enhance peripheral blood hematopoietic stem cell output in patients failing previous mobilizations. Less frequently, plerixafor is used to rescue an unsatisfactory mobilization following chemotherapy (CT) and G-CSF. This study investigates if pre-collection factors affect the CD34+ cell harvest in chemotherapy and G-CSF mobilizations rescued by plerixafor. Clinical and hematological data relative to patients, mobilization, and apheresis products were retrospectively examined. The outcome was completing a target cell dose ≥ 2 × 10^6^ CD34+ cells/kg at first apheresis. The effect exerted on the outcome by patient- and disease-related factors was investigated by univariate and multivariate logistic regression analysis. The analysis included data from 42 patients affected by hematological (39 patients) and non-hematological malignancies (three patients). Twenty-nine patients (69%) attained the target cell dose at first apheresis. Twelve out of the remaining 13 patients received an additional plerixafor administration, and all accomplished the transplant dose at a second apheresis procedure. Day -1 CD34+ PB count (OR1.46, 95% CI 1.1–1.9, *p* = 0.008) and platelet count (OR1.0, 95% CI 1.0–1.0, *p* = 0.033) predicted the achievement of the target dose at first apheresis, independently of pre-mobilization CT, radiation therapy, and disease status at mobilization. At ROC curve analysis, the best cut-off value predicting the successful collection at first apheresis was 7.5/µL for Day -1 CD34+ cell count (AUC 0.830, 0.69 sensitivity, and 0.92 specificity) and 75 × 10^9^/L for Day -1 platelet count (AUC = 0.736, 0.65 sensitivity and 0.85 specificity). In conclusion, on-demand plerixafor rescue allows a successful stem cell collection, irrespectively of disease type and status, prior CT lines, and radiation exposure. Pre-apheresis CD34+ cells and platelet count predict the need for one or two aphereses.

## 1. Introduction

The use of autologous stem cell transplantation (ASCT) following high-dose chemotherapy is a pillar of treatment of patients with various hematological malignancies [1,2]. Over the years, the use of this therapeutic option has been consolidated also in plasma cell disorders other than multiple myeloma (MM), solid tumors such as neuroblastoma or germinal tumors, and autoimmune diseases [3,4,5,6,7]. In candidates for ASCT, apheresis procedures should secure a minimum cell dose of 2 × 10^6^ CD34+ cells/kg [8,9]. Doses below this threshold are associated with delayed recovery of neutrophils and platelets, prolonged transfusion dependency and lower survival rates [10,11,12,13]. Indeed, the optimization of hematopoietic progenitor cell (HPC) mobilization from bone marrow (BM) to peripheral blood (PB) is critical to maximize the apheresis yield. Conventional regimens for HSC mobilization rely on G-CSF as single agent or in combination with chemotherapy (CT) [14]. Nonetheless, failure rates between 10 and 40% with traditional strategies have been reported [15,16,17,18]. Indeed, different criteria have been variably adopted to identify patients with proven or predicted poor mobilization [19,20,21].

Plerixafor is a chemokine receptor antagonist, which acts to prevent the interaction between stromal-derived factor 1 in the bone marrow (BM) niche and C-X-C chemokine receptor type 4 on HPCs, thereby promoting their migration from BM to PB [22]. Mobilization regimens including plerixafor alone or in combination have been described in autologous and allogeneic transplant settings [23,24]. When used as up-front mobilization, the combination of G-CSF and plerixafor significantly expands the proportion of MM and lymphoma patients achieving a satisfactory cell dose collection [25,26]. In addition to the up-front administration, plerixafor is used in many patients in the course of chemotherapy mobilization, based on a scarce CD34+ cell concentration before apheresis, or when the apheresis failed to achieve an adequate HPC dose (the so called ‘rescue’ or ‘just in time’ use) [18,23]. This strategy reduces the number of mobilization failures and maximizes the cell yield in a significant part of poor mobilizer patients [27,28,29,30,31].

When plerixafor is used as an upfront strategy, clinical variables related to the disease status and previous chemo- or radiotherapy can influence the success of collection [32]. Nevertheless, it remains to be elucidated whether these variables might predict mobilization also when plerixafor is used as a rescue of CT and G-CSF mobilization. In order to rationalize therapies and considering the high costs of plerixafor, it could be worth predicting the collection failure before starting plerixafor administration. In this study, we investigated poor mobilizer patients receiving plerixafor as a rescue after G-CSF and CT mobilization. The aim of the study was to identify which factors, among those usually associated with poor mobilization, predict the failure to collect a successful transplant dose.

## 2. Materials and Methods

### 2.1. Study Design

The study is a retrospective, single center, cohort study including consecutive patients undergoing PBSC collection with a plerixafor-containing mobilizing regimen at Fondazione Policlinico Universitario Agostino Gemelli IRCCS. Patients were accrued between February 2018 and December 2021. Plerixafor was administered in agreement with guidelines incorporated into local procedures [33]. The study was conducted according to the Declaration of Helsinki and received the approval from the Ethics Committee of Fondazione Policlinico A. Gemelli IRCCS (prot.0030921/20; study ID 3326).

### 2.2. Mobilization Protocol and Apheresis Procedures

Chemotherapy regimens consisted of cyclophosphamide (4 g/m^2^) in patients with MM and plasma cell leukemia and disease-specific chemotherapy (MiCMA regimen in lymphomas [34], and other platin- or cytarabine-based regimens in further patients) [14]. G-CSF was administered at 5 μg/kg/day after chemotherapy completion, starting at different days depending on the CT regimen. All patients included in the study showed an expected peak of CD34+ cells in concomitance with a WBC count recovery below 20/μL (Day -1). Therefore, they were considered poor mobilizers [20] and received plerixafor at the dose of 240 μg/kg/day [33]. Leukaphereses were carried out starting after 8–10 h from plerixafor (Day 1). Patients failing to achieve the transplant dose of 2 × 10^6^ CD34+ cells/kg at first apheresis were given an additional plerixafor administration, and a second apheresis was performed (Day 2). All procedures were performed using the COBE Spectra or Spectra Optia continuous flow cell separators (Terumo BCT, Shinagawa, Tokyo) with the mononuclear cell collection program and a ratio of anticoagulant to blood of 1:12. The anticoagulant always consisted of sodium citrate solution (Fresenius Kabi, Bad Homburg, Germany). In all patients, 2.5–3 total blood volumes (TBV, defined as the processing blood volume divided by patient’s blood volume) were processed.

### 2.3. Collected Data and Definitions

Clinical data included basal demographics (age, gender, and body weight), diagnosis, disease status at apheresis, type and number of prior chemotherapy regimens, previous radiotherapy, and red blood cell (RBC) or platelet (PLT) transfusions after chemotherapy administration. Laboratory data included complete cell blood count and CD34+ cell count at Day -1 and on the day of collection. Collection data included blood volume processed, content of total nucleated cells (TNC), and content of CD34+ cells in the apheresis product. Complete cell count and CD34+ cell enumeration were performed on peripheral blood and apheresis samples as previously reported [35]. Therapy response was assessed using the Revised Response Criteria for Malignant Lymphoma [36] and International Myeloma Working Group Uniform Response Criteria [37]. For the study purpose, patients were divided into those that achieved any type of response and those with stable/progressive disease.

### 2.4. Study Objectives and Definitions

The primary study objective was to identify variables predicting a target cell dose ≥ 2 × 10^6^ CD34+ cells/kg at first apheresis. The secondary objective was to identify variables predicting the target cell dose with the second apheresis in patients who failed the primary outcome.

### 2.5. Statistical Analysis

Continuous variables were expressed as median (interquartile range, IQR) and categorical variables as n, (%). Differences between variables were assessed by the Mann–Whitney U test for continuous variables and Fisher’s exact test for categorical variables. The Wilcoxon test was used to compare paired data. The effect of different variables on the outcome was evaluated in univariate and multivariate logistic regression analysis. For multivariate analysis, the backward stepwise method was used, and the quality of the model was assessed through the test of Hosmer and Lemeshow. Results were expressed as odds ratio (OR) with relative 95% confidence intervals (CI). The cut-off best discriminating the outcome was identified by Receiving Operating Characteristics (ROC) curve analysis, according to the highest sensitivity and specificity. Linear regression analysis was used to predict the CD34+ cell yield concentration according to other variables. Differences with *p* < 0.05 were considered significant. Analyses were performed using the IBM SPSS Statistics for Windows (Version 27.0. IBM Corp. Released 2020, Armonk, NY, USA) and GraphPad Prism (version 6.00 for Windows, GraphPad Software, La Jolla, CA, USA). The data supporting the findings of this study are available from the senior author upon reasonable request.

## 3. Results

Forty-two consecutive patients were included in the study. Among them, 19 had plasma cell disorders (16 MM, two POEMS and one plasma cell leukemia), 18 patients had refractory or relapsed Hodgkin’s or non-Hodgkin’s lymphoma, two had germ cell tumors, and three were affected by other types of malignancies (medulloblastoma, acute myeloid leukemia, and cerebral histiocytosis). Patients included in this study underwent 74 total apheresis: Table 1 shows demographics, clinical characteristics and the yield of CD34+ cells collected at Day -1 and day-2 apheresis. The two patients with germ cell tumors had been included in a previous study [38].

According to the diagnosis, a different number of transplant doses were planned: lymphoma patients were candidates to collect one single dose, whilst two or more transplants were planned for MM patients, depending on to the patient age. To investigate the study outcome (i.e., the collection of a target CD34+ cell dose ≥ 2 × 10^6^ /kg), we included in the analysis 54 total procedures: 42 were performed on day 1, and 12 were performed on day 2. Among the first day-apheresis, the target dose was achieved in 29 (69%). Twelve out of 13 patients failing the outcome at first apheresis received a second plerixafor administration and all of them completed the target dose at the second apheresis. The remaining patient had a previous CD34+ cell collection stored and did not undergo further apheresis.

### 3.1. Variables Predicting the Completion of the Target Dose at First Apheresis

Table 2 illustrates the association between target dose completion at first apheresis and different characteristics related to patients and disease.

Table 3 displays the analysis of clinical and laboratory parameters associated with the completion of the CD34+ cell dose at first apheresis.

**Figure 1:**
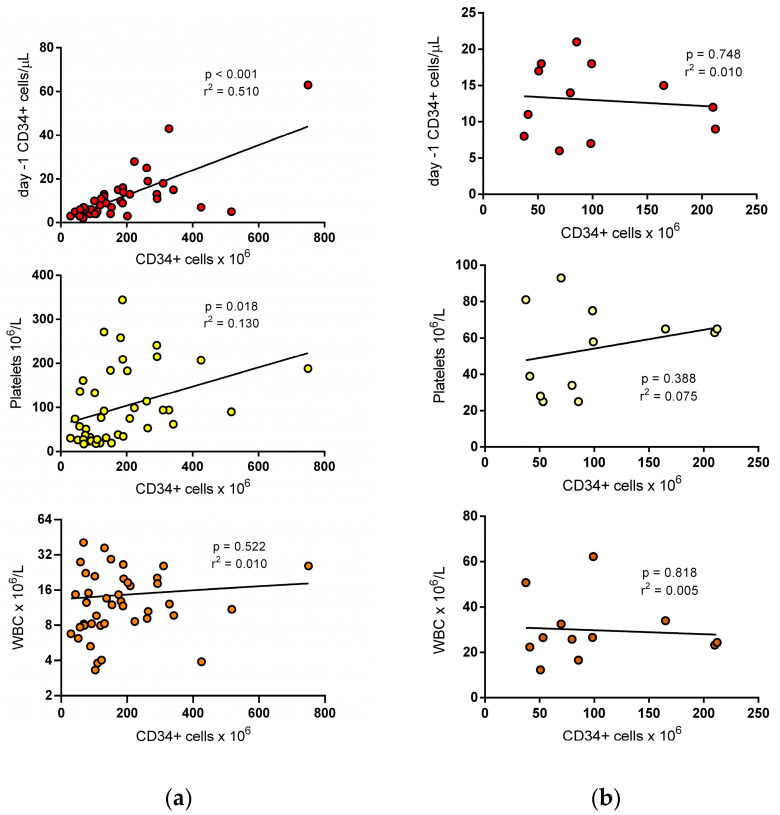
Correlation between CD34+ cell yield (normalized to 10 L of blood volume processed) and parameters recorded on Day -1. (**a**) The CD34+ cell yield at first collection showed a significant correlation with pre-apheresis CD34+ cell count and, to a lesser extent, with platelet count; there was no correlation with WBC count. (**b**) The CD34+ cell yield obtained at the second procedure showed no correlation with CD34+ cells, platelets, and WBC count recorded on the previous day.

At univariate analysis, basal demographics, body weight, number of pre-mobilization CT lines (>3), previous lenalidomide, RBC or PLT transfusions were not associated with CD34+ cell dose achievement. Patients with lymphomas less frequently than others achieved the target dose (OR 0.20, 95% CI 0.05–0.80, *p* = 0.026), while previous radiation therapy and the response status seemed not to have a significant impact. Among laboratory data, only Day -1 PB CD34+ cell concentration (OR 1.46, 95% CI 1.10–1.95, *p* = 0.008) and platelet count (OR 1.01, 95% CI 1.00–1.03, *p* = 0.033) were significantly associated with the successful collection (Table 2). At ROC curve analysis of Day -1 CD34+ cell concentration, the best cut-off to predict the primary outcome was 7.5/µL (AUC = 0.830 ± 0.06, *p* < 0.000, with a sensitivity of 0.69 and a specificity of 0.93). For Day -1 platelet count, the best cut-off value was 75 × 10^9^/L (AUC = 0.736 ± 0.07, *p* = 0.015, with a sensitivity of 0.65 and a specificity of 0.85). At multivariate analysis, both these parameters retained their significance (Table 3). Overall, the OR for achieving the transplant dose in a single collection was 26.6 (95% CI 2.9–237.4) for patients with Day -1 CD34 + cells ≥ 7.5/µL, 10.4 (95% CI 1.9–56.6) for patients with platelet count ≥ 75 × 10^9^/L, and 20.8 (95% CI 3.8–110.3) for those with both findings. The CD34+ cell yield at first apheresis (normalized to 10 L of blood volume processed), showed a significant correlation with Day -1 CD34+ cell count (r2 0.510, *p* < 0.001) and, to a lesser extent, with platelet count (r2 0.130, *p* = 0.018), whilst there was no correlation with white blood cell (WBC) count (Figure 1a).

### 3.2. Variables Predicting the Completion of the Target Dose at Second Apheresis

Thirteen patients failed the primary outcome. One patient had no further collection, whilst 12 underwent a second plerixafor administration with subsequent apheresis. There was no significant difference between CD34+ cell yields of first and second apheresis (1.25, IQR 0.95–1.40 versus 1.35, IQR 1.25–2.75; *p* = 0.211 at the Wilcoxon test). All patients successfully completed the target dose. In contrast to the first collection, the CD34 yield (normalized to 10 L of blood volume processed) of the second procedure showed no correlation with CD34+ cell and platelet count recorded on the previous day (Figure 1b). As expected, the CD34 + cell yield was proportional to the CD34+ cell count recorded on the same morning (r^2^ 0.846, *p* < 0.001).

## 4. Discussion

Plerixafor is widely used in patients failing an adequate PBSC mobilization. Acknowledged risk factors for poor or suboptimal mobilization after chemotherapy and G-CSF include age >60 years, progressive disease, severe bone marrow involvement, previous administration of specific drugs or radiotherapy [8,14,18,39]. However, the relevance of the same factors when a poor mobilization is rescued by on-demand plerixafor is not clear.

In this retrospective cohort study, we primarily investigated which pre-collection factors could predict a satisfactory CD34+ cell yield (i.e., a dose ≥ 2 × 10^6^ CD34+ cells/kg) in patients receiving plerixafor on-demand as a rescue therapy during a poor mobilization after chemotherapy and G-CSF. Our analysis shows that peripheral blood CD34+ cells on Day -1 is the most reliable independent parameter to predict the collection of the target dose in a single apheresis session. Conversely, we observed no effect of gender, disease status, number of pre-mobilization CT lines, and previous exposure to lenalidomide or radiation therapy. The CD34+ cell count in the PB is used to monitor stem cell mobilization and schedule the most appropriate collection time. Peripheral CD34+ cell count has been correlated with the number of collected CD34+ cells since the first seminal works on autografting [10,11,40]. An age over 60 and/or a previous myelosuppressive chemotherapy and/ or a spike in circulating stem cells of less than 20 cells CD34+/µL are accepted indications for plerixafor administration [41]. Accordingly, the position statement of the European Group for Blood and Marrow Transplantation states that a pre-apheresis CD34+ cell count >20 is sufficient to start collection, while if the count is <5 a sufficient collection is unlikely [33]. Our study provides evidence that a cut-off >7.5 CD34+ cells/µL is an acceptable threshold to reach a successful collection with a single dose of plerixafor. Moreover, in our series of patients, those failing the primary outcome could achieve the target dose after the second plerixafor administration.

Notably, most data on plerixafor have been gathered in patients who had previously failed chemotherapy mobilization and were subsequently re-mobilized with the up-front combination of plerixafor and G-CSF [18,23]. However, our findings suggest that on-demand plerixafor administration in association with chemotherapy can be a valid strategy, in particular for patients requiring chemotherapy for the disease control, likewise allowing them to access a potential curative option as ASCT. In this study, plerixafor on-demand rescued poor mobilization in practically all patients, preventing further up-front re-mobilizations and simplifying the schedule of ASCT. Albeit with limits connected to the retrospective design, these observations are relevant to the real-life management of poor-mobilizer patients.

Ishii et al. developed a quantitative model to predict CD34+ cell yield after up-front plerixafor and G-CSF administration. The model incorporates the CD34+ cell count on the day before the apheresis, the number of prior chemotherapy regimens, and the disease status [32]. Even if CD34+ cell count correlated significantly with collection yield on the first day, the coefficient of determination was very low and was remarkably improved by adding the other variables [32]. At variance with Ishii et al., our data are gathered in patients also receiving chemotherapy. Overall, our results suggest this strategy might overcome the negative effects on mobilization exerted by disease status at mobilization, previous radiotherapy or number of chemotherapy lines.

Our results also suggest that platelet count might predict an efficient mobilization. The connection between platelet count and CD34+ cell output has been previously reported when plerixafor is used as up-front mobilization therapy. Lanza et al. and Bakeer et al. reported a baseline platelet count of 140,000/µL and of 153,000/µL as predicting factors of a good response to plerixafor, respectively [42,43]. In the present study, a platelet threshold of 75,000/µL seems to predict a successful collection in one single apheresis. All these findings may be collectively explained by the role of megakaryocyte in supporting the CD34+ cell expansion in the hematopoietic niche [44].

We observed that patients with lymphoma had a lower successful rate at first apheresis than patients with other diagnoses. This finding was not confirmed at multivariate analysis. Nevertheless, other studies reported that plerixafor (either with G-CSF alone or in association with chemotherapy) elicits a lower rate of collection of sufficient transplant doses and lower CD34+ cell yield in patients with non-Hodgkin lymphoma compared to patients with either MM or HL [27,28,29,39]. This is not surprising, considering that lymphoma patients, at variance with MM, usually experience greater hematological toxicity before ASCT due to different chemotherapy lines. In addition, the more frequent involvement of BM in comparison with HL may underlie this finding.

## 5. Conclusions

Mobilization failure may pose a risk to the therapeutic program of patients who are candidates for ASCT. Scheduling an additional mobilization course not only causes the transplant delay but also might convey additional toxicity. Moreover, in cases needing a central vein catheter for stem cell collection, organizing supplementary mobilization and apheresis procedures further complicates patient management from a logistic point of view. Using Plerixafor to rescue chemotherapy and G-CSF mobilization at risk of failure offers a practical solution to prevent these issues. A CD34+ cell count > 7.5/µL seems sufficient to achieve a minimum transplant dose in one single apheresis. According to our experience, practically all poor-mobilizer patients can achieve a transplant dose with a second Plerixafor administration, allowing them to access ASCT.

## Figures and Tables

**Table 1:** Characteristics of 42 investigated patients and 74 overall apheresis.

**Basal demographics**	Age at apheresis (years)	58.9 (49.7–63.3)
	Males (%)	31 (74)
	Body weight (kg)	73.5 (61.5–85)
**Diagnosis**	Hodgkin/non-Hodgkin Lymphoma	3/15 (7/36)
	Multiple Myeloma/Plasma Cell Leukemia/POEMS	16/2/1 (38/5/2)
	Others ^a^	5 (12)
**Disease status**	Any response	37 (88)
	Stable disease/progressive disease	5 (12)
**Prior therapies**	1 chemotherapy line	20 (48)
	2 chemotherapy lines	16 (38)
	≥3 chemotherapy lines	6 (14)
	Lenalidomide-containing regimen	7 (17)
	Radiation therapy	5 (12)
**Patients needing transfusions ^b^**	RBC	6 (14)
	Platelets	11 (26)
**Procedures**	Day 1 apheresis	42 (100)
	Day 2 apheresis	12 (29)
**CD34+ cell yield (10^6^/kg)**	Day 1 apheresis	2.8 (1.4–4.3)
	Day 2 apheresis	2.6 (1.5–4.0)
**Total blood volume processed ^c^**	Day 1 apheresis	2.75 (2.47–2.94)
	Day 2 apheresis	2.79 (2.40–2.98)

Continuous variables are given as median (interquartile range). Categorical variables are given as a number (%). ^a^: Other diagnoses included germ cell tumor (two patients), medulloblastoma, acute myeloid leukemia, and cerebral histiocytosis. ^b^: number of patients receiving RBC or PLT units during mobilization. ^c^: Total blood volume is defined as the amount of blood processed/patient blood volume.

**Table 2:** Clinical and laboratory characteristics of patients grouped according to the completion of the target cell dose ≥ 2 × 10^6^ CD34+ cells/kg at first apheresis.

CD34+ Cells Yield ≥ 2 × 10^6^/kg	Yes (n = 29)	No (n = 13)
Age at apheresis, years	58 (49–63)	60 (50–64)
Sex (male) (%)	21 (72)	10 (77)
Lymphoma (%)	9 (31)	9 (69)
Multiple myeloma (%)	14 (48)	2 (15)
Responsive disease (%)	26 (90)	11 (85)
Prior chemotherapy regimens ≥ 3 (%)	4 (14)	2 (15)
Previous lenalidomide (%)	6 (21)	1 (8)
Prior radiation (%)	5 (17)	0
Day -1 CD34+ cells/µL	11.0 (6.5–12.5)	5.0 (3.5–6.0)
Day -1 WBC count (10^9^/L)	12.1 (8.8–19.2)	8.2 (7.2–21.6)
Day -1 hemoglobin (g/dL)	11.2 (9.1–12.1)	10.3 (9.3–11.2)
Day -1 platelet count (10^9^/L)	94 (36–197)	32 (25–65)
Previous RBC transfusions (%)	4 (14)	2 (15)
Previous PLT transfusions (%)	5 (17)	6 (46)
Total blood volume processed	2.7 (2.4–3.0)	2.7 (2.5–2.9)
CD34+ cell yield (×10^6^/kg) at first apheresis	3.3 (2.6–5.6)	1.2 (1.0–1.4)

Continuous variables are given as median (interquartile range). Categorical variables are given as number (%). Total blood volume is defined as the amount of blood processed/patient blood volume.

**Table 3:** Association between clinical and laboratory parameters and completion of the CD34+ cell dose at first apheresis. Results of univariate and multivariate analyses are shown.

CD34+ Cells Yield ≥ 2 × 10^6^/kg	Univariate		Multivariate	
	OR (95% CI)	*p* Value	OR (95% CI)	*p* Value
Age at apheresis, years	0.98 (0.94–1.03)	0.460		NS
Sex, female versus male	0.79 (0.17–3.62)	0.759		NS
Body weight, kg	0.98 (0.95–1.02)	0.349		NS
Lymphoma diagnosis	0.20 (0.05–0.82)	0.026		NS
Responsive disease	0.63 (0.09–4.34)	0.64		NS
Number of prior chemotherapy regimens ≥ 3	1.14 (0.18–7.15)	0.892		NS
Previous lenalidomide	3.13 (0.34–29.09)	0.316		NS
Day -1 CD34+ cells/µL	1.47 (1.10–1.95)	0.009		NS
Day -1 WBC count (10^9^/L)	1.00 (1.00–1.00)	0.978	2.06 (1.06–4.08)	0.037
Day -1 hemoglobin (g/dL)	1.19 (0.79–1.76)	0.399		NS
Day -1 platelet count (10^9^/L)	1.01 (1.01–1.02)	0.033	1.04 (1.00–1.07)	0.049
Previous RBC transfusions	0.88 (0.14–5.53)	0.892		NS
Previous PLT transfusions	0.24 (0.57–1.04)	0.57		NS
Total blood volume processed	1.37 (0.22–8.41)	0.733		NS

Radiation therapy was recorded in five patients achieving the target dose and was not investigated at regression analysis.

## Data Availability

The data that support the findings of this study are available from the corresponding author upon reasonable request.

## References

[1] Passweg J.R., Baldomero H., Chabannon C., Basak G.W., de la Cámara R., Corbacioglu S., Dolstra H., Duarte R., Glass B., Greco R. (2021). Hematopoietic Cell Transplantation and Cellular Therapy Survey of the EBMT: Monitoring of Activities and Trends over 30 Years. Bone Marrow Transplant..

[2] D’Souza A., Fretham C., Lee S.J., Arora M., Brunner J., Chhabra S., Devine S., Eapen M., Hamadani M., Hari P. (2020). Current Use of and Trends in Hematopoietic Cell Transplantation in the United States. Biol. Blood Marrow Transplant..

[3] Kim Y.R. (2022). Update on the POEMS Syndrome. Blood Res..

[4] Vaxman I., Dispenzieri A. (2021). The Role of Autologous Stem Cell Transplantation in Amyloidosis. Oncology.

[5] Haghiri S., Fayech C., Mansouri I., Dufour C., Pasqualini C., Bolle S., Rivollet S., Dumas A., Boumaraf A., Belhout A. (2021). Long-Term Follow-up of High-Risk Neuroblastoma Survivors Treated with High-Dose Chemotherapy and Stem Cell Transplantation Rescue. Bone Marrow Transplant..

[6] Pierantoni F., Maruzzo M., Bimbatti D., Finotto S., Marino D., Galiano A., Basso U., Zagonel V. (2022). High Dose Chemotherapy Followed by Autologous Hematopoietic Stem Cell Transplantation for Advanced Germ Cell Tumors: State of the Art and a Single-Center Experience. Crit. Rev. Oncol. Hematol..

[7] Ramalingam S., Shah A. (2021). Stem Cell Therapy as a Treatment for Autoimmune Disease-Updates in Lupus, Scleroderma, and Multiple Sclerosis. Curr. Allergy Asthma Rep..

[8] Giralt S., Costa L., Schriber J., DiPersio J., Maziarz R., McCarty J., Shaughnessy P., Snyder E., Bensinger W., Copelan E. (2014). Optimizing Autologous Stem Cell Mobilization Strategies to Improve Patient Outcomes: Consensus Guidelines and Recommendations. Biol. Blood Marrow Transplant..

[9] Duong H.K., Savani B.N., Copelan E., Devine S., Costa L.J., Wingard J.R., Shaughnessy P., Majhail N., Perales M.A., Cutler C.S. (2014). Peripheral Blood Progenitor Cell Mobilization for Autologous and Allogeneic Hematopoietic Cell Transplantation: Guidelines from the American Society for Blood and Marrow Transplantation. Biol. Blood Marrow Transplant..

[10] To L.B., Dyson P.G., Juttner C.A. (1986). Cell-Dose Effect in Circulating Stem-Cell Autografting. Lancet.

[11] Weaver C.H., Hazelton B., Birch R., Palmer P., Allen C., Schwartzberg L., West W. (1995). An Analysis of Engraftment Kinetics as a Function of the CD34 Content of Peripheral Blood Progenitor Cell Collections in 692 Patients after the Administration of Myeloablative Chemotherapy. Blood.

[12] Bensinger W., Appelbaum F., Rowley S., Storb R., Sanders J., Lilleby K., Gooley T., Demirer T., Schiffman K., Weaver C. (1995). Factors That Influence Collection and Engraftment of Autologous Peripheral-Blood Stem Cells. J. Clin. Oncol..

[13] Pérez-Simón J.A., Martín A., Caballero D., Corral M., Nieto M.J., Gonzalez M., Vazquez L., López-Berges C., Cañizo M.C., Mateos M.V. (1999). Clinical Significance of CD34+ Cell Dose in Long-Term Engraftment Following Autologous Peripheral Blood Stem Cell Transplantation. Bone Marrow Transplant..

[14] Sheppard D., Bredeson C., Allan D., Tay J. (2012). Systematic Review of Randomized Controlled Trials of Hematopoietic Stem Cell Mobilization Strategies for Autologous Transplantation for Hematologic Malignancies. Biol. Blood Marrow Transplant..

[15] Namdaroglu S., Korkmaz S., Altuntas F. (2017). Management of Mobilization Failure in 2017. Transfus. Apher. Sci..

[16] Steinberg M., Silva M. (2010). Plerixafor: A Chemokine Receptor-4 Antagonist for Mobilization of Hematopoietic Stem Cells for Transplantation after High-Dose Chemotherapy for Non-Hodgkin’s Lymphoma or Multiple Myeloma. Clin. Ther..

[17] Pusic I., Jiang S.Y., Landua S., Uy G.L., Rettig M.P., Cashen A.F., Westervelt P., Vij R., Abboud C.N., Stockerl-Goldstein K.E. (2008). Impact of Mobilization and Remobilization Strategies on Achieving Sufficient Stem Cell Yields for Autologous Transplantation. Biol. Blood Marrow Transplant..

[18] Chen J., Lazarus H.M., Dahi P.B., Avecilla S., Giralt S.A. (2021). Getting Blood out of a Stone: Identification and Management of Patients with Poor Hematopoietic Cell Mobilization. Blood Rev..

[19] Wuchter P., Ran D., Bruckner T., Schmitt T., Witzens-Harig M., Neben K., Goldschmidt H., Ho A.D. (2010). Poor Mobilization of Hematopoietic Stem Cells-Definitions, Incidence, Risk Factors, and Impact on Outcome of Autologous Transplantation. Biol. Blood Marrow Transplant..

[20] Olivieri A., Marchetti M., Lemoli R., Tarella C., Iacone A., Lanza F., Rambaldi A., Bosi A. (2012). Proposed Definition of “poor Mobilizer” in Lymphoma and Multiple Myeloma: An Analytic Hierarchy Process by Ad Hoc Working Group Gruppo ItalianoTrapianto Di Midollo Osseo. Bone Marrow Transplant..

[21] Olivieri J., Attolico I., Nuccorini R., Pascale S.P., Chiarucci M., Poiani M., Corradini P., Farina L., Gaidano G., Nassi L. (2018). Predicting Failure of Hematopoietic Stem Cell Mobilization before It Starts: The Predicted Poor Mobilizer (PPM) Score. Bone Marrow Transplant..

[22] Uy G.L., Rettig M.P., Cashen A.F. (2008). Plerixafor, a CXCR4 Antagonist for the Mobilization of Hematopoietic Stem Cells. Expert Opin. Biol. Ther..

[23] Bilgin Y.M. (2021). Use of Plerixafor for Stem Cell Mobilization in the Setting of Autologous and Allogeneic Stem Cell Transplantations: An Update. J. Blood Med..

[24] Romon I., Castillo C., Cid J., Lozano M. (2022). Use of Plerixafor to Mobilize Haematopoietic Progenitor Cells in Healthy Donors. Vox Sang..

[25] DiPersio J.F., Stadtmauer E.A., Nademanee A., Micallef I.N.M., Stiff P.J., Kaufman J.L., Maziarz R.T., Hosing C., Früehauf S., Horwitz M. (2009). Plerixafor and G-CSF versus Placebo and G-CSF to Mobilize Hematopoietic Stem Cells for Autologous Stem Cell Transplantation in Patients with Multiple Myeloma. Blood.

[26] DiPersio J.F., Micallef I.N., Stiff P.J., Bolwell B.J., Maziarz R.T., Jacobsen E., Nademanee A., McCarty J., Bridger G., Calandra G. (2009). Phase III Prospective Randomized Double-Blind Placebo-Controlled Trial of Plerixafor plus Granulocyte Colony-Stimulating Factor Compared with Placebo plus Granulocyte Colony-Stimulating Factor for Autologous Stem-Cell Mobilization and Transplantation for Patients with Non-Hodgkin’s Lymphoma. J. Clin. Oncol..

[27] Lor K.W., Helmons P.J., Belew H., Lane J.R., Ball E.D. (2012). Plerixafor as First- and Second-Line Strategies for Autologous Stem Cell Mobilization in Patients with Non-Hodgkin’s Lymphoma or Multiple Myeloma. Pharmacotherapy.

[28] Hübel K., Fresen M.M., Salwender H., Basara N., Beier R., Theurich S., Christopeit M., Bogner C., Galm O., Hartwig R. (2011). Plerixafor with and without Chemotherapy in Poor Mobilizers: Results from the German Compassionate Use Program. Bone Marrow Transplant..

[29] Hübel K., Fresen M.M., Apperley J.F., Basak G.W., Douglas K.W., Gabriel I.H., Geraldes C., Jaksic O., Koristek Z., Kröger N. (2012). European Data on Stem Cell Mobilization with Plerixafor in Non-Hodgkin’s Lymphoma, Hodgkin’s Lymphoma and Multiple Myeloma Patients. A Subgroup Analysis of the European Consortium of Stem Cell Mobilization. Bone Marrow Transplant..

[30] Milone G., Conticello C., Leotta S., Michieli M.G., Martino M., Marco A.L., Di Spadaro A., Cupri A., Condorelli A., Milone G.A. (2020). Plerixafor On-Demand in Association with Low-Dose Cyclophosphamide and G-CSF in the Mobilization of Patients with Multiple Myeloma: High Effectiveness, Low Toxicity, and Affordable Cost. Leuk. Res. Rep..

[31] Vaxman I., Muchtar E., Jacob E., Kapoor P., Kumar S., Dispenzieri A., Buadi F., Dingli D., Gonsalves W., Kourelis T. (2021). The Efficacy and Safety of Chemotherapy-Based Stem Cell Mobilization in Multiple Myeloma Patients Who Are Poor Responders to Induction: The Mayo Clinic Experience. Transplant. Cell. Ther..

[32] Ishii A., Jo T., Arai Y., Oshima S., Kanda J., Kitawaki T., Matsui K., Niwa N., Nakagawa Y., Takaori-Kondo A. (2022). Development of a Quantitative Prediction Model for Peripheral Blood Stem Cell Collection Yield in the Plerixafor Era. Cytotherapy.

[33] Mohty M., Hübel K., Kröger N., Aljurf M., Apperley J., Basak G.W., Bazarbachi A., Douglas K., Gabriel I., Garderet L. (2014). Autologous Haematopoietic Stem Cell Mobilisation in Multiple Myeloma and Lymphoma Patients: A Position Statement from the European Group for Blood and Marrow Transplantation. Bone Marrow Transplant..

[34] Ortu La Barbera E., Chiusolo P., Laurenti L., Menichella G., Di Febo A.L., Piccirillo N., Sora F., Marra R., Teofili L., Leone G. (2000). MiCMA: An Alternative Treatment for Refractory or Recurrent Hodgkin’s Disease. Ann. Oncol..

[35] Teofili L., Chiusolo P., Valentini C.G., Metafuni E., Bellesi S., Orlando N., Bianchi M., Giammarco S., Sica S., Bacigalupo A. (2020). Bone Marrow Haploidentical Transplant with Post-Transplantation Cyclophosphamide: Does Graft Cell Content Have an Impact on Main Clinical Outcomes?. Cytotherapy.

[36] Cheson B.D., Pfistner B., Juweid M.E., Gascoyne R.D., Specht L., Horning S.J., Coiffier B., Fisher R.I., Hagenbeek A., Zucca E. (2007). Revised Response Criteria for Malignant Lymphoma. J. Clin. Oncol..

[37] Kumar S., Paiva B., Anderson K.C., Durie B., Landgren O., Moreau P., Munshi N., Lonial S., Bladé J., Mateos M.V. (2016). International Myeloma Working Group Consensus Criteria for Response and Minimal Residual Disease Assessment in Multiple Myeloma. Lancet Oncol..

[38] Corbingi A., Metafuni E., Di Salvatore M., Putzulu R., Chiusolo P., Schinzari G., Massini G., Rossi E., Zini G., Cassano A. (2022). Successful “on-Demand” Plerixafor for Autologous Peripheral Blood Stem-Cells Transplantation for Relapsed/Refractory Germ Cell Tumors. J. Clin. Apher..

[39] Lee K.H., Jung S.K., Kim S.J., Jang J.H., Kim K., Kim W.S., Jung C.W., Kim D.W., Kang E.S. (2014). Incidence and Risk Factors of Poor Mobilization in Adult Autologous Peripheral Blood Stem Cell Transplantation: A Single-Centre Experience. Vox Sang..

[40] Armitage S., Hargreaves R., Samson D., Brennan M., Kanfer E., Navarrete C. (1997). CD34 Counts to Predict the Adequate Collection of Peripheral Blood Progenitor Cells. Bone Marrow Transplant..

[41] Summary of Product Characteristics. https://www.ema.europa.eu/en/documents/product-information/mozobil-epar-product-information_en.pdf.

[42] Lanza F., Lemoli R.M., Olivieri A., Laszlo D., Martino M., Specchia G., Pavone V., Imola M., Pasini A., Milone G. (2014). Factors Affecting Successful Mobilization with Plerixafor: An Italian Prospective Survey in 215 Patients with Multiple Myeloma and Lymphoma. Transfusion.

[43] Bakeer M., Zubair A.C., Roy V. (2020). Low Baseline Platelet Count Predicts Poor Response to Plerixafor in Patients with Multiple Myeloma Undergoing Autologous Stem Cell Mobilization. Cytotherapy.

[44] De Graaf C.A., Kauppi M., Baldwin T., Hyland C.D., Metcalf D., Willson T.A., Carpinelli M.R., Smyth G.K., Alexander W.S., Hilton D.J. (2010). Regulation of Hematopoietic Stem Cells by Their Mature Progeny. Proc. Natl. Acad. Sci. USA.

